# Influence of visual feedback, hand dominance and sex on individuated finger movements

**DOI:** 10.1007/s00221-021-06100-0

**Published:** 2021-04-19

**Authors:** Anna-Maria Johansson, Helena Grip, Louise Rönnqvist, Jonas Selling, Carl-Johan Boraxbekk, Andrew Strong, Charlotte K. Häger

**Affiliations:** 1 Department of Psychology, Umeå University, Umeå, Sweden; 2 Department of Community Medicine and Rehabilitation, Physiotherapy, Umeå University, Umeå, Sweden; 3 Department of Radiation Sciences, Umeå University, Umeå, Sweden; 4 Danish Research Centre for Magnetic Resonance (DRCMR), Centre for Functional and Diagnostic Imaging and Research, Copenhagen University Hospital Hvidovre, Hvidovre, Denmark; 5 Institute of Sports Medicine Copenhagen (ISMC), Copenhagen University Hospital Bispebjerg, Copenhagen, Denmark

**Keywords:** Finger movements, Vision, Motor control, Kinematics, Sex differences, Spatiotemporal

## Abstract

The ability to perform individual finger movements, highly important in daily activities, involves visual monitoring and proprioception. We investigated the influence of vision on the spatial and temporal control of independent finger movements, for the dominant and non-dominant hand and in relation to sex. Twenty-six healthy middle-aged to old adults (M age = 61 years; range 46–79 years; females *n* = 13) participated. Participants performed cyclic flexion–extension movements at the metacarpophalangeal joint of one finger at a time while keeping the other fingers as still as possible. Movements were recorded using 3D optoelectronic motion technique (120 Hz). The movement trajectory distance; speed peaks (movement smoothness); Individuation Index (II; the degree a finger can move in isolation from the other fingers) and Stationarity Index (SI; how still a finger remains while the other fingers move) were extracted. The main findings were: (1) vision only improved the II and SI marginally; (2) longer trajectories were evident in the no-vision condition for the fingers of the dominant hand in the female group; (3) longer trajectories were specifically evident for the middle and ring fingers within the female group; (4) females had marginally higher II and SI compared with males; and (5) females had fewer speed peaks than males, particularly for the ring finger. Our results suggest that visual monitoring of finger movements marginally improves performance of our non-manipulative finger movement task. A consistent finding was that females showed greater independent finger control compared with males.

## Introduction

Successful interaction with, and manipulation of, objects requires adequate control of the movements and forces of individual fingers (Schieber and Santello 2004). The Individuation Index (II) is a measure of how independently a given finger can move from the other fingers of the same hand, while the related Stationarity Index (SI) estimates how still the finger can remain when the other fingers of the same hand move (Häger-Ross and Schieber 2000). Using these types of measures, albeit previously with 2D motion analysis, control of individual fingers has been shown to be limited by both mechanical coupling between the fingers and restrictions in neuromuscular control (Beisteiner et al. 2001; Dechent and Frahm 2003; Lang and Schieber 2004; Lotze et al. 2000; Schieber 2001; Schieber and Hibbard 1993). Independent finger movements are limited by a partly overlapping network of muscles in the hand (Lang and Schieber 2004; Lemon 1999; Schieber 1995) and studies show that mechanical coupling between the fingers is greatest for the index, middle and ring finger, with the thumb being the most individuated finger (Häger-Ross and Schieber 2000; Lang and Schieber 2004). Active neuromuscular control, however, appears to mainly limit the movements of the ring and little finger, especially if the range of motion for the produced movements is large (Lang and Schreiber 2004).

The ability to move fingers with a high degree of independence is important in everyday functional dexterity (Schieber and Santello 2004). A body of evidence shows, as expected, reduced capacity to move the fingers independently in neurological disorders including stroke (e.g., Lang and Schieber 2004; Raghavan et al. 2006). In persons without known neuromuscular disability, investigations have focused on the neuromuscular control of individuated finger movements with vision of their fingers allowed during task performance. Only pilot data in abstract format have so far been reported questioning the potential effects of vision on the independence of finger control (Hayes and Schieber 1996). Hence there are good reasons to investigate this issue further and add to the literature by evaluating the role vision of the fingers has on such performance.

Multimodal monitoring of actions is crucial during dexterous tasks. For example, visual as well as proprioceptive information from mechanoreceptors in the skin (Johansson and Flanagan 2008), joints, and muscles (Dimitriou and Edin 2010; see Proske and Gandevia 2012 for a review) of the hand and fingers are integrated and used to estimate position and positional change during action (Saunders and Knill 2004). Visual monitoring of the hands and fingers during dexterous common activities has been shown to be frequent, predictive and related to object manipulation (Land et al. 1999). When visual feedback of the hand and fingers is omitted, the kinematics of reaching and grasping behavior typically change (Churchill et al. 2000; Jakobson and Goodale 1991; Jeannerod 1984; Karl et al. 2012). Such visual monitoring of the hands during goal-directed movements is automatic and enables corrections to be made in the motion path (e.g., Fourneret and Jeannerod 1998; Land et al. 1999; Pélisson et al. 1986). However, the role of visual feedback when making individuated finger movements in a non-manipulative movement task remains to be established.

Skilled dexterous tasks in general show substantial differences related to hand dominance, with the dominant hand having higher proficiency than the non-dominant hand. However, documented side differences in performance of individual finger movements is rare, with the exception of tapping tasks that have identified increased finger-tapping speed, less speed variability and stronger and more focused myoelectric activity in the dominant index finger (Heuer 2007). Previous studies investigating the ability to produce isolated finger forces at the distal phalanx (Reilly and Hammond 2000) and in finger motion trajectories (Häger-Ross and Schieber 2000) have not detected any considerable effect of hand dominance. However, it is not known whether omitting visual control over the fingers movements (i.e., limiting the feedback about positional change solely to internal sources) would reveal a hand dominance effect. That said, notable side differences have been found in motor control during goal-directed tasks performed with the index finger where velocity-based movement control outcome measures show faster and greater control of the index finger of the dominant right hand compared to that of the non-dominant left (Aoki Rivlis and Schieber 2016). Thus, it is possible that more pronounced side differences in the control of individual finger movement emerges in tasks with higher complexity, i.e., goal-directed tasks. Another possibility is that side differences are not readily detected in studies using individuation index calculations as it is a measure of individual finger movement control relative to the other fingers of the hand. Hence, analyzing temporal aspects of the movement of each finger could offer further insight into side differences in the control of specific fingers. In goal directed movements, sudden changes in tangential velocity, either indicated by acceleration or velocity changes, are to be interpreted as an online corrective response to perceived errors in the trajectory (see Khan et al. 2006 for an extensive review). To summarize, we anticipate both increased finger individuation and smoother, less variable outcomes in conditions when participants have vision of their hands. We also hypothesize that participants will have smoother finger movements with their dominant side regardless of which finger is being moved compared with the concomitant finger of the non-dominant side.

Another aspect under investigation in the current study was whether the sex of the participant influences performance of the finger individuation task. Sex differences in dexterity are well-documented in childhood (e.g., Moser and Reikerås 2016) and also in young to middle adulthood (Rohr 2006; Ruff and Parker 1993). When forced to choose between speed and accuracy, adult males have been shown to favor speed over accuracy, while females prioritize accuracy at the expense of speed (Rohr 2006). Further, a finding that appears to be stable from the late teen years until retirement age is that females perform better at fine manipulation eye-hand coordination tasks (Purdue Pegboard Test) than males; whereas, males outperform females on tasks that require speed production, such as finger tapping (Ruff and Parker 1993). Similarly, older males (60–88 years of age) were slower in the Purdue Pegboard Test compared to females of the same age (Vasylenko Gorecka and Rodríguez-Aranda 2018). This sex difference was however not found for younger participants (19–38 years of age) (Vasylenko et al. 2018). Increased manipulation skills and better accuracy for females than males have been proposed due to their increased engagement throughout life in tasks that require fine manipulation skills, consequently gaining more experience and a higher level of expertise than males. However, it has been suggested (Vasylenko et al. 2018) that sex differences in fine manipulation tasks could be due to hand size differences between males and females.

To summarize, the purpose of the present study was threefold. First, the main aim was to extend earlier findings of finger synergies by investigating the effect of having vision of the hands during task performance on measures of movement control over individual fingers in healthy middle-aged to older adults using 3D optoelectronic motion capture. To accomplish this research goal, previously developed measures of finger individuation (Häger-Ross and Schieber 2000) were extended to 3D kinematics and a velocity-based jerk measure (speed peaks) was developed to encompass both spatial and temporal aspects of finger movement control during the flexion and extension phases of movements. Second, as the effect of hand preference in non-manipulative finger control tasks remains unclear, we evaluated the potential effect of hand dominance on these outcomes and an additional measure of movement trajectory length. Third, we aimed to extend the sparse existing evidence of possible sex differences on finger motor control by comparing females and males on the movement control outcomes while controlling for hand size. We anticipated increased finger individuation and smoother, less variable outcomes during visual feedback and that participants would have smoother finger movements with their dominant hand than the corresponding finger of the non-dominant hand.

## Methods

### Participants

Twenty-seven (females *n* = 13) participants 46–79 years of age (*M* = 61.4; SD = 8.2) without known musculoskeletal or neurologically-derived movement problems were recruited through advertisements, colleagues and acquaintances. All had normal or corrected-to-normal vision. One female participant aged 64 was excluded due to measurement errors resulting in a total of 26 participants included in the analyses (Table 1, participant demographics). The study was approved by the Regional Ethical Review Board in Umeå (dnr 2011-199-31 M) and conducted in accordance with the Declaration of Helsinki.

**Table 1:** Participant demographics and any self-reported fine motor skills

	Age (y)			Handedness	Self-reported fine motor skills*
	*M*	SD	Range	(R/L)	(Y/N)
Female (*n* = 12)	61.5	10.4	46–79	12/0	9/3
Male (*n* = 14)	61.7	6.7	49–69	13/1	6/8

*n* number, *y* years, *M* mean, *SD* standard deviation, *R* right, *L* left, *Y* yes, *N* no. * Self-reported fine motor skills, for example: cabinetry; fly tying; piano, guitar, or trumpet playing; typing; crochet; sewing; knitting; a profession that requires fine motor manipulation

### Apparatus and procedure

#### Task setup

**Fig. 1:**
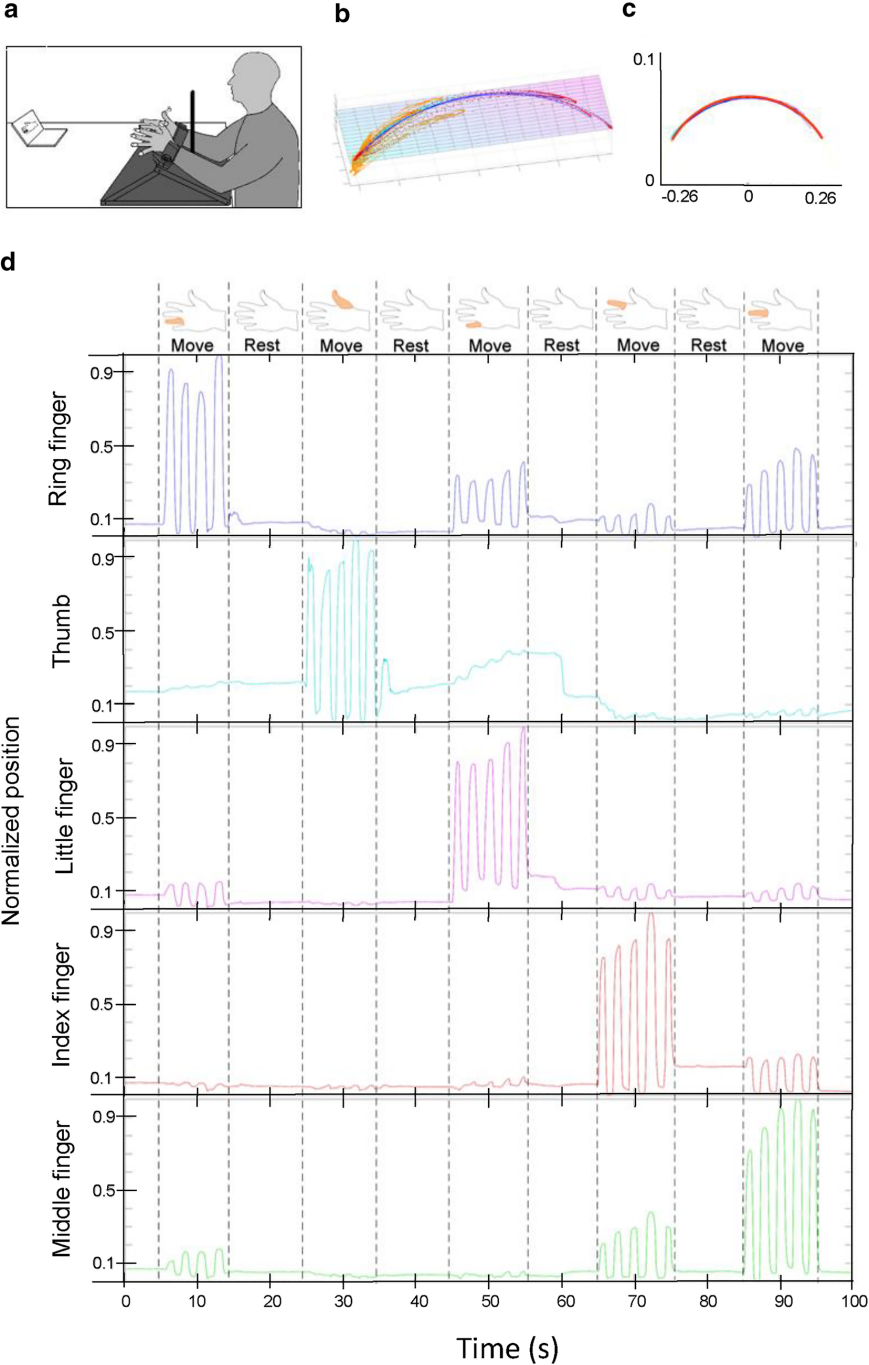
**a** Experimental set-up with an example sequence in the no-vision condition, in the vision condition the screen occluding vision of the hands was removed. **b** The 3D data of a movement trajectory of one finger in the total movement interval is fitted to a plane that can be rotated for optimum fit. **c** Projection of converted 3D data to a 2D arc shape showing the circular fit. **d** Example of the instructions and movement sequence provided for the participants during the test (images in the top panel) and associated normalized finger movements during the experiment for each digit. The *x* axis shows duration from 0 to 100 s

The ability to perform individualized finger movements with or without visual feedback of the fingers was assessed in a unimanual task. Participants were instructed to perform cyclic extension–flexion movements at the metacarpophalangeal joint, keeping the active finger straight and the other fingers straight and as still as possible. During performance of the task, participants sat upright with their arms in approximately 45° of flexion, the glenohumeral joint in about 10° of abduction, and the elbow in about 90° of flexion. The wrists were extended about 10° and fixated to a wooden frame with support for the forearms (Fig. 1a). On each fingertip a 7 mm passive reflective marker was affixed with double-sided adhesive tape and the movements were recorded with an optoelectronic system (ProReflex, Qualisys, Sweden; 6 cameras, 120 Hz). For half of the trials, vision of the hands was blocked for the participants by a screen that was placed in front of them (Fig. 1a). Graphical instructions were provided on a computer screen showing which finger that was to be moved. The instructions consisted of a black outline drawing of a hand on a white background where the specific finger that was to be moved was highlighted in red (see Fig. 1a, d). A drawing of a left hand was used for the left hand conditions and a right hand for right hand conditions. Each highlighted finger was shown for 10 s followed by a 10 s rest period. The participants were instructed to move the corresponding finger to that highlighted in the stimuli during the period it was displayed at a self-selected pace. In the rest period the participants were instructed to keep their fingers still in an extended position. For each trial, all five fingers were active once in a randomized order. Each block had a duration of 100 s. Two blocks were performed with each hand for both the vision and no-vision condition, resulting in eight blocks in total. The stimuli presentation was synchronized with the 3D measurement and was programmed in E-prime (version 2; Psychology Software Tools). Starting with vision or no-vision was counterbalanced between participants.

### Data processing

#### Identification of finger movement intervals

Qualisys Track Manager software was used to identify marker trajectories, with an automatic gap-fill of 20 frames. Marker data was then processed (Visual3D v5, C-Motion Inc., Germantown, MD, USA) and filtered (6 Hz 4th-order low-pass zero-phase Butterworth filter). The 10 s total movement interval of each finger during the 100 s block was identified from the velocity profile by first identifying the time windows where finger movements were expected and then the onset and offset of movement. Automatized algorithms identified events according to the following: the criterion for the onset was the frame where the tangential velocity reached 5% of the peak tangential velocity within the identified movement interval. To eliminate false onsets being identified the criteria defined that the onset frame had to be related to a following increase in the tangential velocity reaching a minimum of 15% of the tangential peak velocity. The criterion for offset was the last frame of the movement interval where the tangential velocity reached 5% of the tangential peak velocity. For an offset frame to be identified, the adjacent prior tangential velocity had to reach at least 15% of the tangential peak velocity. Each total movement interval for each finger within the block, as well as the onset and offset events, were visually inspected. Errors were corrected manually by inspecting related graphs of fingertip trajectory (X, Y, Z and normalized planes), speed profiles and the animated 3D movements.

#### Extracted kinematic parameters

The analyses were performed in Visual 3D, and a MATLAB plug-in (MATLAB R2017a, The MathWorks, Inc., Natick, Massachusetts, United States) was constructed for Individuation and Stationarity Index calculations. The 3D finger coordinate data within the identified total movement intervals were subject to kinematic analyses.

### Individuation and Stationarity Index

Based on the normalized positional change of the fingers during task performance, we calculated two indices ranging from 0 to 1, as devised by Häger-Ross and Schieber (2000): (1) Individuation Index (II) quantifying the independence of each individual finger, where 1 indicates complete independence; (2) Stationarity Index (SI) where 1 indicates that the finger remains still when the other fingers move. This is described extensively in Häger-Ross and Schieber (2000), but briefly, the normalized movement trajectory of each digit is calculated (“1” defines maximal finger flexion and “0” defines minimum finger extension). The II is then calculated as 1 minus the average of the correlation slopes of the non-instructed digits, relative to the instructed digit. The SI was calculated as 1 minus the average relative motion slope of that digit during the movement intervals for which it was a non-instructed digit. Further development was made in the current study by employing 3D data instead of 2D data (cf. Häger-Ross and Schieber 2000). This enabled selection of the plane that the finger movements were projected onto. Hence, prior to the calculations of II and SI, a movement plane was fitted to the 3D data within each total movement interval and using a robust fit. Data were then projected onto the movement plane, converting it to a 2D arc shape, then further projected onto a circular fit of that arc. The plane was inspected and corrected if necessary. The data were then transformed to 1D by calculating the distance along the arc to every point from the point of maximum extension. Data were then normalized so that maximum extension was set to 0 and maximum flexion was set to 1 (see Fig. 1b–d for an illustration of data extraction). Finally, SI and II were calculated.

### Speed peaks

To gain additional information about the movement control of each finger, the speed peaks of every flexion and extension phase were extracted and used as a measure of movement smoothness. This was done by identifying local tangential velocity maxima within each phase. Peaks considered as noise, measurement noise and biological noise such as higher frequency tremors were removed by the 6 Hz low-pass filter. The phase where speed peaks were retrieved was identified as follows: the point of maximum flexion and maximum extension within each normalized flexion–extension displacement trajectory was first identified. The dwell phases, when the finger movement changed from extension to flexion or vice versa, occurring around maximum flexion and extension (dwell_flex and dwell_ext) were excluded to avoid the inclusion of minor aberrant speed peaks. The beginning of the dwell phase around peak flexion (dwell_flex) was defined as the point where 95% of the total normalized distance towards maximum flexion had been covered. The end of the dwell phase was defined as the point where the normalized trajectory distance towards maximum extension exceeded 5% of the distance. The dwell phase around maximum extension (dwell_extension) was defined similarly. Flexion and extension phases with missing data were excluded from further analyses. The total number of speed peaks during the 10 s total movement interval for each finger, including both the extension and the flexion phase, was counted and then divided by the number of flexion–extension movements performed within the 10 s total movement interval. The mean value was used in the statistical analyses.

### Movement trajectory distance

The movement trajectory distance in millimeters of each finger was identified in the 2D circular fitted data, using the time points of maximum extension and maximum flexion, respectively. As finger length and marker placement varied between the participants, the movement trajectory distance was only used in analyses of matched pairs.

### Movement frequency

To calculate the movement frequency (MF), we extracted the duration of (defined as the time between the identified onset and offset frames) and the number of extension–flexion movements within the total movement interval of each finger. The MF was then calculated by dividing the number of extensions–flexions within the total movement interval with the total movement interval duration, thus providing the number of cycles/second.

### Statistical analyses

**Fig. 2:**
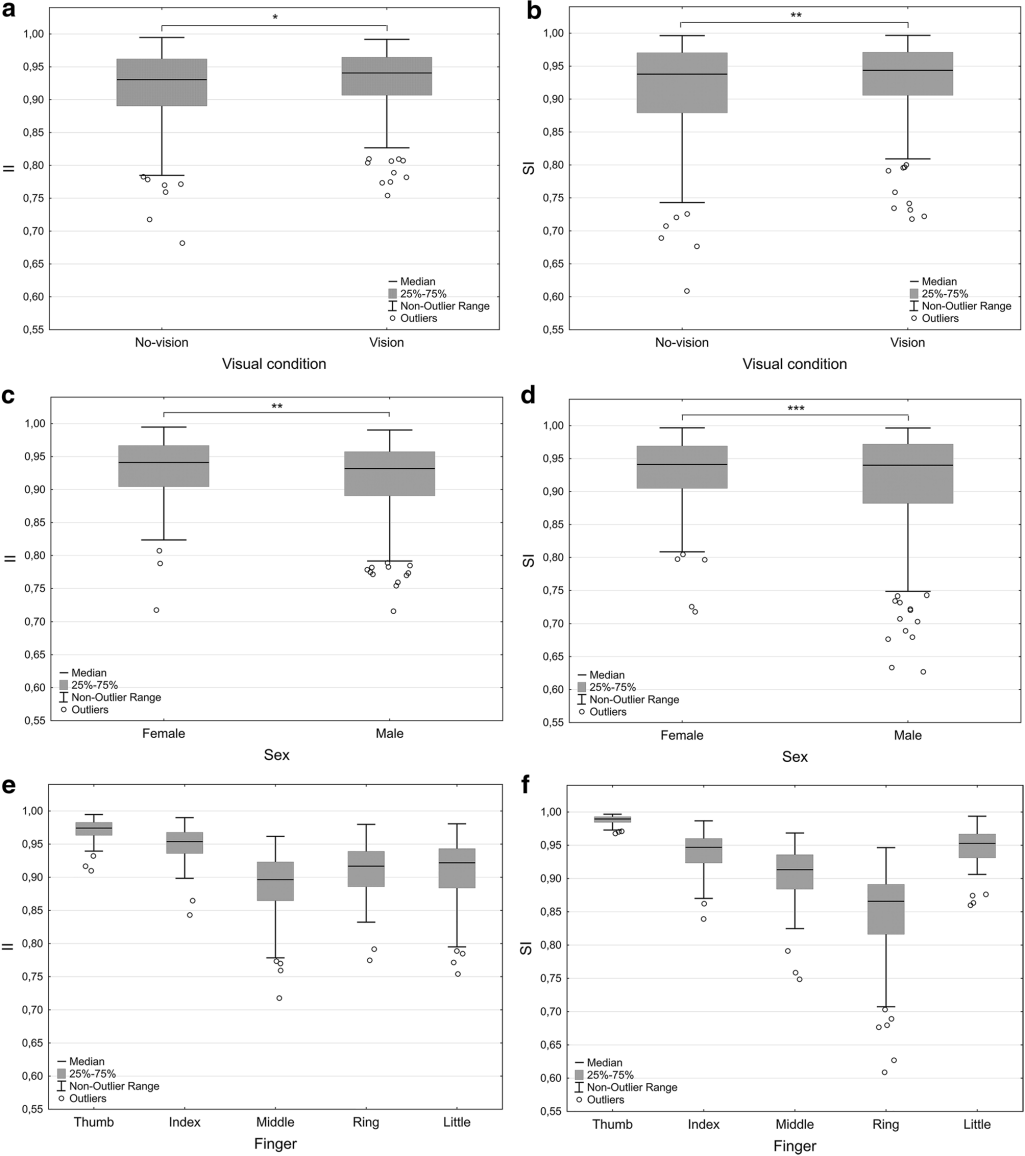
**a**–**f** Illustration of Individuation Index (II) by finger and sex in a the vision and no-vision condition; c divided by sex and e for each of the fingers. Stationarity Index (SI) is shown in b the vision and no-vision condition; **d** by sex, and **f** for the fingers. Lines denote medians, the boxes represent the 25th–75th percentiles, whiskers show the non-outlier range (coefficient of 1), circles show outliers (coefficient of 1.5). Significant main effects are shown in **a**–**d** (* = *p* < 0.05; ** = *p* < 0.01; *** = *p* < 0.001). The main effects for finger were significant (*p* < 0.000000) and significant differences between the fingers (**e** and **f**) were noted for nearly all comparisons. See “Results” section for specifics. The graph depicts original data

**Fig. 3:**
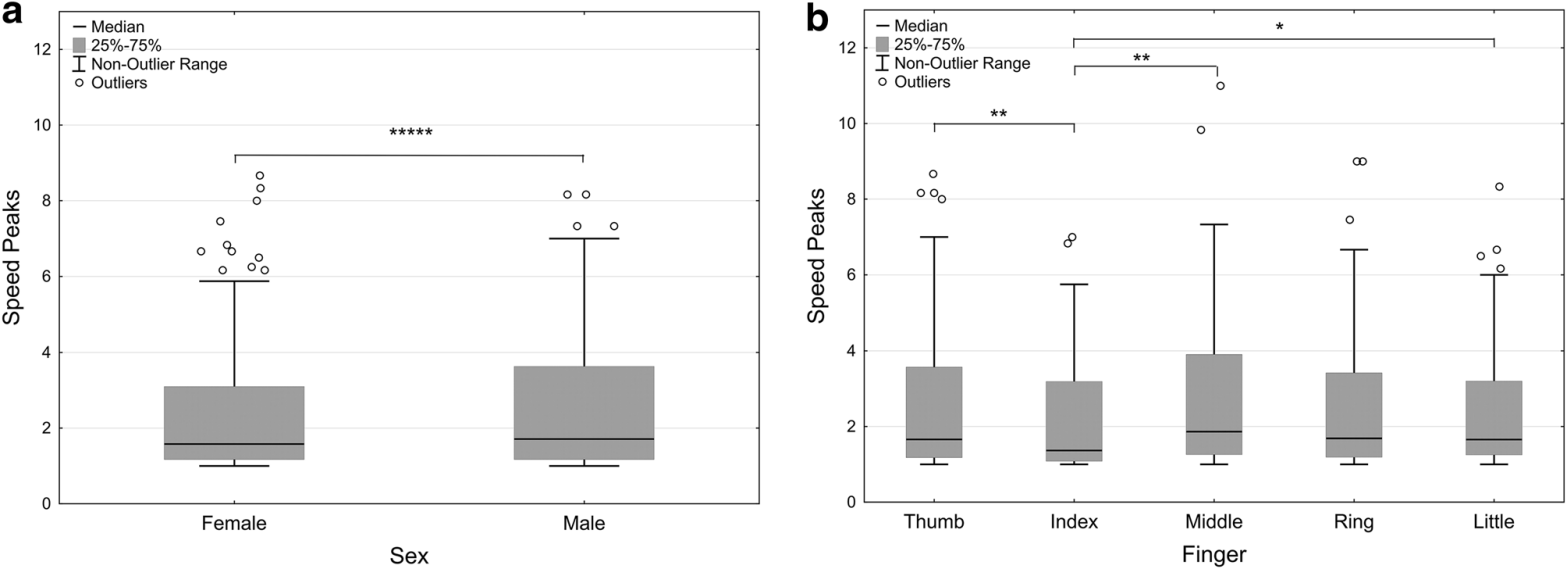
**a**, **b** Illustration of speed peaks for **a** the male and female participants and **b** each finger. Lines denote medians, the boxes represent the 25th–75th percentiles, whiskers show the non-outlier range (coefficient of 1), circles show outliers (coefficient of 1.5). Significant main effects are indicated (* = *p* < 0.05; ** = *p* < 0.01; ***** = *p* < 0.00001). The graph depicts original data

To improve normality in the distribution of the index values (II and SI), their data were transformed according to (ln(1-x)) (denoted _t1-ln_) where ln is the natural logarithm, MF was transformed using ln (denoted _tln_) and speed peaks through the Box–Cox procedure (denoted _tBoxCox_). After transformation, all levels of these outcome measures were normally distributed according to Kolmogorov–Smirnov tests (all *p* values > 0.05). Movement speed has been shown in previous studies to be associated with finger independence measures (Häger-Ross and Schieber 2000) and with measures of jerkiness (e.g., Morasso, Ivaldi, and Ruggiero 1983). Hence, MF was used as a continuous covariate in the II and speed peak analyses. To control for potential effects of finger and hand metrics, we first obtained relevant measures by drawing the contours of the hands of all participants on paper. The length of the middle finger (tip of finger to center of finger base identified by drawing a line from the lowest point between the middle finger and its adjacent fingers and identifying the center of that line) was divided by the width of the hand over the metacarpophalangeal joints (index finger to little finger). The finger/hand metric was used as a covariate in the II, SI and speed peak analyses. For exploratory purposes, Pearson’s correlation was used to assess the influence of MF on the II and speed peaks and of the finger/hand metric on II, SI and speed peaks. The *p* values for each analysis were Bonferroni corrected (0.05/9), yielding a corrected alpha value of 0.0055. The MF and finger/hand metrics were normally distributed. Differences between visual condition (vision vs. no-vision), side (dominant vs. non-dominant hand), sex (female vs. male) and finger (thumb, index, middle, ring and little) were tested by three separate 2 (visual condition) × 2 (side) × 2 (sex) × 5 (finger) analysis of covariance (ANCOVA). Outcome variables were II, SI and speed peaks. Note that figures showing II, SI and speed peaks (Figs. 2a–f and 3a, b) depict original data and include median, range (25–75 percentiles), non-outlier range (coefficient of 1), and outliers (coefficient of 1.5), thus, they are not based on the statistical analyses.

Wilcoxon’s matched pairs tests were used to test side differences of movement trajectory distance. In total, seventy-four tests were performed and thus *p* values were adjusted according to the Bonferroni procedure (0.05/74 = 0.00068). Pearson’s *r* was used as an effect size measure. Outcomes in the ANCOVA analyses were considered significant if the alpha value was < 0.05. Significant interaction effects between any of the categorical predictors and finger were followed-up for each finger with pairwise comparisons (one-way ANOVA, ANCOVA where appropriate). Follow-up analyses were Bonferroni corrected for the number of comparisons made (0.05/5), giving an alpha value for statistical significance of 0.01. Significant main effects for finger were followed-up with Tukey’s Honestly Significant Difference post-hoc tests with a Bonferroni corrected (0.05/20) alpha value for statistical significance of 0.0025. Partial eta square (*η*_p_^2^) was used as effect size estimate. None of the assumptions for the statistical tests were violated.

## Results

### Background analyses

#### Age differences

To check that no systematic difference in age was present between the sexes that could influence our results, a Mann–Whitney *U* test was performed testing for group differences. Results showed no significant age differences (*U* = 81; *p* = 0.897) between the female (Mdn = 60 years) and male (Mdn = 63.5 years) groups. Further, potential associations between age and the outcome variables were tested where no significant correlations were apparent (all *p* values > 0.05).

#### Correlations between the outcome variables and covariates

The only significant correlation between MF_tLog_ and II _t1-ln_ was found for the non-dominant hand (*r* = 0.20; *p* = 0.002). In contrast, significant negative correlations with high *r*’s were found for SP_tBoxCox_ on the main level and within all subgroup splits (visual condition; side; sex; and finger. See Table 2 for specifics) showing a consistent association between increasing MF and a decreasing number of speed peaks. The finger/hand metric did not have any significant correlations with II_t1-ln_, SI_t1-ln_ nor SP_tBoxCox_.

**Table 2:** Correlations between II, MF, MF and speed peaks, II and hand/finger metric, SI and hand/finger metric, and speed peaks and hand/finger metric on original and, where appropriate, transformed data, split by visual condition, dominant and non-dominant side, sex and finger

Visual condition	Side	Sex	Finger	II and MF	II_t1-ln_ and MF_tLog_	SP and MF	SP_tBoxCox_ and MF_tLog_	II and f/h metric	II_t1-ln_ and f/h metric	SI and f/h metric	SI_t1-ln_ and f/h metric	SP and f/h metric	SP_tBoxCox_ and f/h metric
Vision	Dominant	Male	Thumb	0.02	− 0.12	− **0.79**	− **0.93**	0.16	− 0.16	− 0.45	0.45	0.04	0.08
			Index	− 0.09	0.12	− **0.78**	− **0.87**	0.24	− 0.25	− 0.04	0.03	− 0.17	− 0.12
			Middle	0.41	− 0.38	− **0.85**	− **0.93**	− 0.03	0.02	0.14	− 0.14	-0.03	0.09
			Ring	0.01	0.09	− **0.81**	− **0.83**	− 0.34	0.34	0.10	-0.11	0.16	0.21
			Little	0.02	− 0.05	− **0.78**	− **0.92**	0.17	− 0.18	− 0.32	0.32	0.02	0.12
		Female	Thumb	0.38	− 0.33	− 0.71	− 0.71	0.53	− 0.53	− 0.21	0.21	− 0.52	− 0.48
			Index	0.38	− 0.42	− 0.65	− **0.75**	0.67	− 0.68	− 0.19	0.19	− 0.65	− 0.59
			Middle	− 0.62	0.55	− 0.73	− **0.82**	0.00	0.00	0.62	− 0.63	− 0.64	− 0.58
			Ring	− 0.12	0.02	− **0.75**	− **0.80**	0.50	− 0.51	0.19	− 0.17	− 0.53	− 0.49
			Little	− 0.73	0.69	− **0.77**	− **0.87**	− 0.03	0.04	0.50	− 0.50	− 0.78	− 0.56
Vision	Non-dominant	Male	Thumb	− 0.29	0.21	− **0.86**	− **0.93**	− 0.13	0.13	− 0.08	0.08	0.25	0.23
			Index	− 0.39	0.34	− **0.84**	− **0.90**	0.29	− 0.28	0.21	− 0.21	0.06	0.10
			Middle	− 0.09	0.04	− **0.89**	− **0.91**	0.29	− 0.30	0.22	− 0.22	0.16	0.27
			Ring	− 0.56	0.53	− **0.82**	− **0.84**	0.25	− 0.24	0.05	− 0.04	0.20	0.22
			Little	− 0.28	0.23	− **0.78**	− **0.80**	− 0.05	0.05	0.27	− 0.27	0.30	0.28
		Female	Thumb	-0.04	0.05	− 0.73	− **0.82**	0.10	− 0.11	0.42	− 0.42	− 0.13	− 0.20
			Index	**0.18**	− 0.19	− 0.68	− **0.82**	0.00	0.00	-0.35	0.35	− 0.17	− 0.30
			Middle	− 0.58	0.51	− 0.71	− **0.81**	− 0.65	0.64	-0.30	0.29	− 0.23	− 0.38
			Ring	− 0.28	0.25	− 0.63	− **0.84**	− 0.06	0.06	-0.54	0.53	− 0.09	− 0.33
			Little	− 0.71	0.69	− 0.68	− **0.81**	− 0.30	0.30	0.08	− 0.08	− 0.14	− 0.44
No-vision	Dominant	Male	Thumb	− 0.01	− 0.42	− 0.61	− **0.85**	− 0.01	0.01	-0.36	0.35	0.10	0.15
			Index	− 0.14	0.19	− 0.63	− **0.90**	0.28	− 0.28	0.01	− 0.01	− 0.06	0.03
			Middle	0.27	− 0.20	− **0.70**	− **0.88**	0.16	− 0.16	0.14	− 0.14	− 0.06	0.07
			Ring	− 0.02	0.02	− 0.64	− **0.78**	− 0.15	0.16	0.25	− 0.24	− 0.04	0.00
			Little	0.03	− 0.11	− 0.63	− **0.81**	0.32	− 0.31	0.16	− 0.16	− 0.09	0.03
		Female	Thumb	0.19	− 0.22	− 0.71	− 0.73	0.14	− 0.14	0.00	0.00	− 0.35	− 0.38
			Index	0.13	− 0.19	− **0.78**	− **0.92**	0.11	− 0.11	− 0.10	0.10	− 0.28	− 0.45
			Middle	-0.39	0.31	− **0.76**	− **0.85**	− 0.14	0.13	0.18	− 0.17	− 0.39	− 0.49
			Ring	− 0.35	0.27	− **0.81**	− **0.87**	0.34	− 0.34	0.14	− 0.13	− 0.48	− 0.46
			Little	− 0.29	0.19	− **0.76**	− **0.83**	0.14	− 0.13	0.27	− 0.27	− 0.32	− 0.48
No-vision	Non-dominant	Male	Thumb	− 0.16	0.14	− 0.65	− **0.85**	0.08	− 0.08	-0.23	0.23	0.24	0.25
			Index	− 0.08	0.07	− 0.64	− **0.84**	0.32	− 0.31	0.18	− 0.18	0.21	0.22
			Middle	0.01	− 0.76	− **0.74**	− **0.88**	0.19	− 0.21	0.29	− 0.27	0.17	0.35
			Ring	− 0.24	0.22	− 0.66	− **0.82**	0.35	− 0.33	0.09	− 0.09	0.22	0.24
			Little	− 0.42	0.37	− 0.67	− **0.76**	0.04	− 0.04	0.33	− 0.33	0.24	0.37
		Female	Thumb	0.56	− 0.51	− 0.73	− **0.85**	− 0.26	0.25	− 0.27	0.27	− 0.06	− 0.07
			Index	− 0.01	− 0.01	− 0.67	− **0.80**	− 0.41	0.40	− 0.59	0.59	− 0.07	− 0.15
			Middle	− 0.44	0.37	− 0.73	− **0.89**	− 0.51	0.49	− 0.56	0.56	− 0.08	− 0.22
			Ring	− 0.47	0.44	− 0.72	− **0.88**	− 0.53	0.54	− 0.49	0.48	− 0.07	− 0.16
			Little	− 0.10	0.02	− 0.66	− **0.85**	− 0.23	0.22	− 0.41	0.41	− 0.06	− 0.29

Bold numbers indicate a significant correlation. *II* Individuation Index, *II*_*t1-ln*_ transformed II, *SP* speed peaks, *SP*_*tBoxCox*_ transformed SP, *MF* movement frequency, *MF*_*tLog*_ transformed MF, *f/h metric* finger/hand metric, *SI* Stationarity Index, *SI*_*t1-ln*_ transformed SI. For specifics in how the transformations were done see the Statistical analysis section. All values are Person’s *r*

### The effects of visual condition, side, sex, and finger

#### Results summary

The main findings were: (1) vision of the hands during task performance led to higher IIs and SIs, although effects were small and data contained large variability (Table 3 and Fig. 2a–f); (2) movement trajectories were longer in the no-vision condition for specifically the dominant hand of the female group; and (3) particularly for the middle and ring fingers (Table 4); (4) females had marginally higher IIs and SIs than males (moderate effects and high variability); (5) females had fewer speed peaks than males, particularly for the ring finger. The thumb and index finger had the highest independence and the index finger had fewer speed peaks than the other fingers.

**Table 3:** Means and standard deviations for main effect comparisons for visual condition, dominant and non-dominant side, sex and finger

	II		SI		Speed peaks	
	*M*	SD	*M*	SD	*M*	SD
**Visual condition**						
** Vision**						
Original	0.928	0.005	0.928	0.064	2.46	1.67
Transformed	1.086	0.055	1.077	0.074	0.50	0.34
** No-vision**						
Original	0.919	0.056	0.920	0.068	2.58	1.87
Transformed	1.076	0.064	1.090	0.080	0.51	0.35
**Side**						
**Dominant**						
Original	0.924	0.049	0.924	0.060	2.57	1.76
Transformed	1.081	0.054	1.081	0.070	0.52	0.34
**Non-dominant**						
Original	0.924	0.056	0.924	0.071	2.47	1.78
Transformed	1.081	0.064	1.082	0.860	0.49	0.35
**Sex**						
**Female**						
Original	0.931	0.045	0.931	0.051	2.46	2.01
Transformed	1.070	0.050	1.070	0.570	0.48	0.35
**Male**						
Original	0.917	0.057	0.918	0.076	2.54	1.79
Transformed	1.090	0.066	1.090	0.090	0.54	0.34
**Finger**						
Thumb						
Original	0.971	0.018	0.988	0.007	2.73	1.92
Transformed	1.030	0.019	1.010	0.007	0.62	0.42
**Index**						
Original	0.946	0.035	0.939	0.034	2.20	1.58
Transformed	1.060	0.038	1.070	0.037	0.40	0.35
**Middle**						
Original	0.889	0.048	0.901	0.058	2.66	1.81
Transformed	1.120	0.055	1.110	0.073	0.61	0.42
**Ring**						
Original	0.907	0.049	0.845	0.071	2.43	1.57
Transformed	1.100	0.057	1.170	0.090	0.54	0.36
**Little**						
Original	0.906	0.055	0.949	0.026	2.60	1.92
Transformed	1.100	0.064	1.050	0.028	0.53	0.33

*II* Individuation Index, *SI* Stationarity Index

**Table 4:** Means and standard deviations for each finger on the outcome variables in the visual and non-visual condition organized by side (dominant and non-dominant) and by sex (female, male)

	Non-dominant side																			
	Male					Female					Male					Female				
	Thumb	Index	Middle	Ring	Little	Thumb	Index	Middle	Ring	Little	Thumb	Index	Middle	Ring	Little	Thumb	Index	Middle	Ring	Little
**Vision**																				
**II**																				
*M*	0.97	0.95	0.89	0.92	0.89	0.97	0.96	0.91	0.91	0.92	0.97	0.94	0.88	0.91	0.90	0.97	0.96	0.90	0.92	0.93
SD	0.01	0.03	0.04	0.03	0.05	0.01	0.03	0.04	0.04	0.05	0.02	0.05	0.05	0.07	0.07	0.01	0.01	0.04	0.03	0.03
**II**_**t1-ln**_																				
*M*	1.03	1.06	1.11	1.08	1.12	1.03	1.04	1.09	1.09	1.08	1.04	1.06	1.13	1.10	1.10	1.03	1.04	1.11	1.08	1.08
SD	0.01	0.04	0.04	0.03	0.06	0.01	0.03	0.04	0.04	0.05	0.02	0.05	0.06	0.09	0.08	0.01	0.01	0.05	0.03	0.04
**SI**																				
*M*	0.99	0.94	0.91	0.83	0.95	0.99	0.95	0.91	0.88	0.95	0.99	0.94	0.90	0.82	0.94	0.99	0.94	0.92	0.88	0.96
SD	0.00	0.03	0.04	0.07	0.02	0.01	0.02	0.03	0.07	0.03	0.00	0.03	0.09	0.09	0.04	0.01	0.03	0.02	0.05	0.02
**SI**_**t1-ln**_																				
*M*	1.01	1.06	1.10	1.19	1.05	1.01	1.06	1.09	1.13	1.05	1.01	1.06	1.12	1.20	1.06	1.01	1.07	1.08	1.13	1.05
SD	0.00	0.03	0.05	0.09	0.02	0.01	0.02	0.04	0.08	0.03	0.00	0.03	0.11	0.12	0.04	0.01	0.03	0.02	0.05	0.02
**MF**																				
*M*	0.74	0.78	0.79	0.73	0.76	0.66	0.75	0.70	0.70	0.69	0.79	0.79	0.78	0.80	0.78	0.73	0.76	0.74	0.72	0.71
SD	0.35	0.31	0.31	0.26	0.29	0.29	0.33	0.27	0.29	0.27	0.27	0.28	0.30	0.28	0.27	0.28	0.31	0.29	0.29	0.29
**MF**_**tln**_																				
*M*	− 0.41	− 0.33	− 0.32	− 0.37	− 0.34	− 0.50	− 0.38	− 0.42	− 0.44	− 0.43	− 0.29	− 0.29	− 0.32	− 0.29	− 0.32	− 0.39	− 0.36	− 0.38	− 0.40	− 0.41
SD	0.49	0.41	0.43	0.37	0.40	0.46	0.45	0.40	0.43	0.40	0.35	0.38	0.41	0.39	0.39	0.40	0.43	0.39	0.42	0.41
** SP**																				
*M*	3.3	2.4	2.8	2.3	2.7	2.8	1.9	2.3	2.3	2.4	2.4	2.2	2.8	2.4	2.6	2.1	1.9	2.2	2.3	2.7
SD	2.4	1.6	1.8	1.3	1.9	2.1	1.3	1.6	1.4	1.6	1.3	1.4	1.8	1.5	1.6	1.5	1.4	1.4	2.1	2.2
**SP**_**tBoxCox**_																				
*M*	0.6	0.5	0.6	0.5	0.6	0.6	0.3	0.5	0.5	0.5	0.5	0.4	0.6	0.5	0.6	0.4	0.3	0.5	0.4	0.5
SD	0.4	0.4	0.3	0.3	0.3	0.4	0.4	0.4	0.4	0.3	0.3	0.4	0.4	0.3	0.3	0.3	0.4	0.3	0.3	0.3
**MTd**																				
*M*	180	173	190	156	154	151	126	142	126	97	173	182	186	162	165	164	145	162	128	92
SD	36	39	51	37	37	34	24	30	31	18	41	32	45	30	49	34	34	46	34	30
**No-vision**																				
**II**																				
*M*	0.97	0.93	0.88	0.90	0.89	0.97	0.95	0.88	0.89	0.92	0.97	0.94	0.88	0.90	0.88	0.98	0.95	0.88	0.91	0.93
SD	0.02	0.04	0.04	0.04	0.05	0.01	0.02	0.06	0.05	0.04	0.03	0.04	0.05	0.07	0.09	0.01	0.02	0.05	0.04	0.03
**II**_**t1-ln**_																				
*M*	1.03	1.08	1.13	1.11	1.12	1.03	1.05	1.12	1.12	1.09	1.03	1.06	1.13	1.11	1.13	1.03	1.05	1.13	1.10	1.07
SD	0.02	0.05	0.05	0.05	0.06	0.01	0.02	0.08	0.05	0.04	0.03	0.04	0.06	0.08	0.10	0.01	0.02	0.06	0.05	0.03
**SI**																				
*M*	0.99	0.93	0.88	0.83	0.94	0.98	0.93	0.90	0.86	0.95	0.99	0.94	0.89	0.81	0.94	0.99	0.93	0.91	0.86	0.96
SD	0.01	0.04	0.05	0.06	0.03	0.01	0.04	0.04	0.06	0.02	0.01	0.04	0.10	0.09	0.03	0.00	0.03	0.04	0.04	0.02
**SI**_**t1-ln**_																				
*M*	1.01	1.07	1.13	1.18	1.06	1.02	1.08	1.11	1.15	1.05	1.01	1.06	1.13	1.22	1.06	1.01	1.07	1.10	1.15	1.04
SD	0.01	0.04	0.06	0.07	0.03	0.01	0.05	0.04	0.07	0.03	0.01	0.05	0.13	0.13	0.03	0.00	0.04	0.04	0.05	0.02
**MF**																				
*M*	0.78	0.78	0.81	0.77	0.80	0.63	0.68	0.67	0.67	0.67	0.79	0.83	0.83	0.83	0.82	0.69	0.72	0.70	0.68	0.67
SD	0.48	0.48	0.44	0.43	0.42	0.25	0.28	0.28	0.27	0.27	0.43	0.46	0.44	0.41	0.40	0.30	0.32	0.33	0.30	0.29
**MF**_**tln**_																				
*M*	− 0.37	− 0.39	− 0.33	− 0.37	− 0.33	− 0.54	− 0.48	− 0.50	− 0.48	− 0.48	− 0.35	− 0.31	− 0.30	− 0.28	− 0.31	− 0.47	− 0.42	− 0.46	− 0.47	− 0.50
SD	0.48	0.53	0.50	0.46	0.49	0.43	0.46	0.46	0.42	0.46	0.49	0.51	0.50	0.46	0.49	0.48	0.47	0.49	0.46	0.47
**SP**																				
*M*	2.9	2.7	2.8	2.6	2.6	2.9	2.5	2.7	2.6	2.5	2.9	2.2	2.7	2.5	2.5	2.4	2.0	2.8	2.5	2.8
SD	1.8	2.1	1.9	1.4	2.2	2.1	2.1	2.2	1.6	1.9	2.1	1.6	1.7	1.7	1.6	2.0	1.6	2.3	1.9	2.7
**SP**_**tBoxCox**_																				
*M*	0.6	0.5	0.6	0.6	0.5	0.6	0.5	0.5	0.5	0.5	0.6	0.4	0.6	0.5	0.5	0.5	0.3	0.5	0.5	0.5
SD	0.4	0.4	0.4	0.3	0.4	0.3	0.4	0.3	0.3	0.3	0.3	0.4	0.4	0.4	0.3	0.4	0.4	0.4	0.4	0.3
**MTd**																				
*M*	171	174	211	162	133	150	144	170	141	107	164	176	212	173	137	161	152	176	140	107
SD	39	40	50	38	35	35	32	41	35	26	26	33	42	31	35	21	32	54	43	22

*II* Individuation Index, *II*_*t1-ln*_ transformed II, *SI* Stationarity Index, *SI*_*t1-ln*_ transformed SI, *MF* movement frequency, *MF*_*tLog*_ transformed MF, *SP* speed peaks, *SP*_*tBoxCox*_ transformed SP, *MTd* movement trajectory distance. For specifics in how the transformations were done see the Statistical analysis section

#### Individuation Index

There was a main effect of visual condition [*F*(1, 478) = 6.5, *p* = 0.0113, *η*_p_^2^ = 0.013], with the fingers having higher IIs in the vision (*M* = 0.928, SD = 0.005; *M*_tln_ = 1.086, SD_tln_ = 0.055) than the no-vision (*M* = 0.919, SD = 0.056; *M*_tln_ = 1.076, SD_tln_ = 0.064) condition (Fig. 2a). There was a main effect of sex [*F*(1, 478) = 165, *p* = 0.001, *η*_p_^2^ = 0.006] where the female group (*M* = 0.931, SD = 0.045; *M*_tln_ = 1.070, SD_tln_ = 0.050) had higher indices than the male group (*M* = 0.917, SD = 0.057; *M*_tln_ = 1.090, SD_tln_ = 0.066; Fig. 2c). A main effect of finger was also apparent [*F*(4, 478) = 58.20, *p* = 0.000000, *η*_p_^2^ = 0.33] with all the fingers differing from one another (all *p* values < 0.001), except for the little and ring fingers (Fig. 2e). The thumb (*M* = 0.971, SD = 0.018; *M*_tln_ = 1.030, SD_tln_ = 0.019) was the finger with the highest IIs, followed by the index finger (*M* = 0.946, SD = 0.035; *M*_tln_ = 1.060, SD_tln_ = 0.038), ring finger (*M* = 0.907, SD = 0.049; *M*_tln_ = 1.100, SD_tln_ = 0.057), little finger (*M* = 0.906, SD = 0.005; *M*_tln_ = 1.100, SD_tln_ = 0.064), and finally the middle finger (*M* = 0.889, SD = 0.048; *M*_tln_ = 1.120, SD_tln_ = 0.055). There were no significant interaction effects. MF (*p* = 0.000096) and the hand finger metric (*p* = 0.024) were significant as covariates. The mean values and standard deviations for all comparisons are shown in Table 3 and the sub-levels of analysis are presented Table 4.

#### Stationarity Index

There was a main effect of vision [*F*(1, 478) = 4.28, *p* = 0.039, *η*_p_^2^ = 0.008] where SIs were higher in the visual (*M* = 0.928, SD = 0.064; *M*_tln_ = 1.077, SD_tln_ = 0.074) condition than in the no-vision (*M* = 0.920, SD = 0.068; *M*_tln_ = 1.086, SD_tln_ = 0.080) condition (Fig. 2b). The effect of sex was significant [*F*(1, 478) = 12.01, *p* = 0.0006, *η*_p_^2^ = 0.026] where females (*M* = 0.931, SD = 0.051; *M*_tln_ = 1.070, SD_tln_ = 0.057) had higher SIs than males (*M* = 0.918, SD = 0.076; *M*_tln_ = 1.089, SD_tln_ = 0.091; Fig. 2d). The effect of finger was also significant [*F*(4, 478) = 120.52, *p* = 0.000000, *η*_p_^2^ = 0.50] where all fingers, with the exception of the index and little finger, differed from one another (all *p* values < 0.0001). The thumb (*M* = 0.988, SD = 0.007; *M*_tln_ = 1.010, SD_tln_ = 0.007) had the highest SIs followed by the little finger (*M* = 0.949, SD = 0.026; *M*_tln_ = 1.050, SD_tln_ = 0.028), the index finger (*M* = 0.938, SD = 0.034; *M*_tln_ = 1.065, SD_tln_ = 0.037), the middle finger (*M* = 0.901, SD = 0.058; *M*_tln_ = 1.106, SD_tln_ = 0.073), and finally the ring finger (*M* = 0.845, SD = 0.072; *M*_tln_ = 1.172, SD _tln_ = 0.090) (see Fig. 2f and Table 3 for full descriptives). The interaction between sex and finger [*F*(4, 478) = 5.49, *p* = 0.00025, *η*_p_^2^ = 0.044] was significant and the follow-up pairwise comparisons showed a sex difference for the SI of the ring finger [*F*(1, 99) = 10.16, *p* = 0.0019, *η*_p_^2^ = 0.09] where the female (*M* = 0.870, SD = 0.054; *M*_tln_ = 1.141, SD_tln_ = 0.064) group had higher indices than the male (*M* = 0.824, SD = 0.078; *M*_tln_ = 1.199, SD_tln_ = 0.101) group. No other finger differences were observed between the sexes. In the main analysis, the hand/finger metrics was not a significant covariate.

#### Speed peaks

The mean number of speed peaks during the flexion and extension movements of the fingers showed a significant effect of sex (*F*(1, 478) = 49.31, *p* = 0.000000, *η*_p_^2^ = 0.09) where the female (*M* = 2.46, SD = 2.01; *M*_tBoxCox_ = 0.48, SD_tBoxCox_ = 0.35) group had fewer speed peaks than the male (*M* = 2.54, SD = 1.79; *M*_tBoxCox_ = 0.54, SD_tBoxCox_ = 0.34) group (Fig. 3a). A main effect was also shown for finger [*F*(4, 478) = 4.29, *p* = 0.002, *η*_p_^2^ = 0.035]. Follow-up analyses showed that the index finger differed from the thumb (*p* = 0.000329); middle (*p* = 0.0011); and little (*p* = 0.0037) finger when controlling for the covariates (Table 3 and Fig. 3b). The thumb (*M* = 2.73, SD = 1.92; *M*_tBoxCox_ = 0.62, SD_tBoxCox_ = 0.42) had the highest amount of speed peaks followed by the middle finger (*M* = 2.66, SD = 1.81; *M*_tBoxCox_ = 0.61, SD_tBoxCox_ = 0.42), little finger (*M* = 2.60, SD = 1.92; *M*_tBoxCox_ = 0.53, SD_tBoxCox_ = 0.33), ring finger (*M* = 2.43, SD = 1.57; *M*_tBoxCox_ = 0.54, SD_tBoxCox_ = 0.36) and finally, with the fewest speed peaks, the index finger (*M* = 2.20, SD = 1.58; *M*_tBoxCox_ = 0.40, SD_tBoxCox_ = 0.35). There were no significant interaction effects. In the main analysis, MF (*p* = 0.000000) and hand/finger metric (*p* = 0.0023) were significant covariates. All mean values and standard deviations are presented in Tables 3, 4.

#### Movement trajectory distance

Analyses of actual movement trajectory distance showed that the fingers moved significantly longer (*T* = 12,043, *p* = 0.00005. *r* = 0.73) in the no-vision (*M* mm = 159; SD = 44.4) than the vision (*M* mm = 154; SD = 44.2) condition. When split by the main predictors (side; sex; finger) a significant difference between vision and no-vision was found for the fingers of the dominant hand (*T* = 2413, *p* = 0.00001. *r* = 0.74), which had longer trajectories in the no-vision (*M* mm = 157; SD = 45.5) than the vision (*M* mm = 151; SD = 43.1) condition. When split by sex, there was a difference between the vision and no-vision condition for the females (*T* = 1604, *p* = 0.000000. *r* = 0.86) who had longer trajectories in the no-vision (*M* mm = 144; SD = 0.041) than vision (*M* mm = 133; SD = 0.039) condition. The males showed no such difference. When split by finger, significant differences were evident for the middle (*T* = 173, *p* = 0.000003. *r* = 0.81; *M* mm vision = 171; SD vision = 0.047; *M* mm no-vision = 194; SD = 0.050) and the ring (*T* = 265, *p* = 0.00019. *r* = 0.76, *M* mm vision = 144; SD vision = 0.036; *M* mm no-vision = 155; SD = 0.039) fingers but not the other fingers. When split by side and sex, the females showed a significant difference between the vision and no-vision condition for the dominant (*T* = 300, *p* = 0.000006. *r* = 0.86) hand with longer movement trajectory distances in the no-vision (*M* mm no-vision = 142; SD no-vision = 0.039) than the vision (*M* mm vision = 129; SD vision = 0.033) condition. No significant differences were evident for the males. When split by side and finger a significant difference between the vision and no-vision condition was shown for the middle finger of the dominant hand (*T* = 22, *p* = 0.000097. *r* = 0.89, *M* mm vision = 168; SD vision = 0.048; *M* mm no-vision = 192; SD no-vision = 0.050), again with longer movement trajectory distances in the no-vision than the vision condition. When split by sex and finger, females showed a difference between vision and no-vision for the ring (*T* = 30, *p* = 0.00061. *r* = 0.94, *M* mm vision = 127; SD vision = 0.002; *M* mm no-vision = 140; SD no-vison = 0.038) and middle (*T* = 24, *p* = 0.00036. *r* = 0.87, *M* mm vision = 152; SD vision = 0.039; *M* mm no-vision = 173; SD = 0.047) fingers. All fingers of the female group had longer movement trajectory distances in the no-vision than the vision condition. The males did not have any statistically significant differences. On the smallest fraction of the predictors (side × sex × finger) no significant differences were evident (see Table 4 for *M* and SD).

## Discussion

The main aims of the study were threefold. First, we wanted to investigate if occluding vision of the hands would influence spatial and temporal control of individualized finger movements compared to when allowing visual monitoring in in a sample of healthy middle to old-age adults. Second, we were further interested in exploring the potential effect on finger movement control of hand dominance and third, if the sex of the participants had an effect on the movement control measures used. Our main findings, in relation to these aims, were that vision of the hands during task performance led to slightly higher Individuation indices (II) and Stationarity indices (SI), although effects were small and data had large variability (Table 3 and Fig. 2a–f). No differences between the dominant and non-dominant hand in the II, SI nor movement smoothness (speed peaks) were detectable. Our third aim was to investigate the effect of sex where we found several effects. Movement trajectories were longer in the no-vision condition for the dominant hand of the female group, particularly for the middle and ring fingers (Table 4), and females had higher IIs and SIs and fewer speed peaks than males. As expected, there were also several differences between the fingers where the thumb and index finger showed the highest individuation and the index finger the least number of speed peaks.

### Influences of vision on the temporal and kinematic variables

In tasks where vision is occluded completely, position and positional change are solely reliant on kinesthetic internal feedback. In our study, vision influenced both the movement of individual fingers (II) and the ability to keep fingers still (SI), indicated by higher IIs and SIs when visual monitoring was allowed. Previous studies have shown a substantial effect of vision on kinematics in prehensile movements (e.g., Churchill et al. 2000; Jakobson and Goodale 1991; Karl et al. 2012; Land et al. 1999) but to our knowledge only one pilot study (Hayes and Scheiber 1996) has examined the effect of visual occlusion on a finger individuation task as the one used here. In contrast to our results they found no effects of vision. The effect sizes of visual condition on the finger individuation measures used in the current study were however small, mean value differences marginal and variability high (see Tables 3, 4 and Fig. 2a–f). Unlike studies of visual importance in prehensile tasks (e.g., Churchill et al. 2000; Jakobson and Goodale 1991; Karl et al. 2012; Land et al. 1999), the finger individuation task used here does not include object contact. It is possible that visual input is of less importance in tasks with no object contact if and when the kinesthetic system is well-functioning, as expected among the current healthy sample. We did however find an effect of visual condition on the movement trajectory distances for the female group, where the fingers of the dominant hands had longer trajectories in the no-vision compared to the visual condition (see Table 4). We know from previous studies by Häger-Ross and Schieber (2000) that there is an association between range of motion of the fingers and the individuation indices, where more extensive finger movements were shown to be associated with increased co-movements of non-instructed fingers. As such, the longer movement trajectory distances of the females could potentially affect finger individuation negatively. This matter is discussed in greater detail below. We also know that visual input is important for error corrections in goal-directed tasks (e.g., Khan and Franks 2000; Pélisson et al. 1986; Saunders and Knill 2004) and theoretically vison would play a similar role in the current task where potential co-movements of non-instructed fingers could be visually detected and motion range could be altered/reduced to limit this co-activity. Nevertheless, our results suggest only a minor influence of vision and the reasons for this should be investigated in greater detail. A suggestion is to include variations in trajectory lengths (short, comfortable and extensive) as an additional condition and add eye-movement registrations synchronized with the movement measuring system to increase understanding of the effects of trajectory length on measures of finger movement control and what participants look at during task performance.

### Hand dominance and finger movement control

Considering the second aim of our study, no significant differences between the dominant and non-dominant hand on the movement control measures used were shown. This is in line with findings from previous studies which have used a similar set-up, where no side differences in finger individuation were found (Häger-Ross and Schieber 2000), nor when distal phalanx finger forces have been measured (Reilly and Hammond 2000). Similarly, there were also no detectable side differences in the number of speed peaks, despite two previous studies showing side differences for velocity-based measures in finger control tasks (Aoki et al. 2016; Heuer 2007). However, it was only the index fingers that were measured in both of those studies and thus their findings are not directly comparable to those presented here. Furthermore, Aoki et al. (2016) measured side differences in a goal-directed tracking task and the study by Heuer (2007) focused on differences in variability between the dominant and non-dominant hands. Although beyond the scope of the current study, including outcomes of single flexion–extension cycles of the index fingers and associated between-cycle variability could be an avenue for further investigations in this matter. In relation to movement trajectory distances significantly longer paths were however found for the fingers of the dominant hand compared to those of the non-dominant hand (6 mm mean difference between the dominant and non-dominant side) when visual monitoring was not allowed. When visual monitoring was allowed during performance, there were no significant differences in trajectory distances between the hands. When fractioning the analyses down to sub-groupings it became clear that the difference between the no-vision and vision condition resided only within the dominant hand of the female group, which indicates a potential influence of sex on our outcome measures.

### Effects of sex in relation to vision and finger control

A number of sex differences were apparent which is interesting considering that the experimental task is non-manipulative and that the possible effect of constitutional and systematic finger/hand differences were controlled for. Differences in hand size has been raised as a concern previously when studying sex differences in dexterity (Vasylenko et al. 2018). Our study showed that the female group was significantly better at performing individual finger movements (higher IIs) and keeping the non-instructed fingers still (higher SIs) than the male group, albeit with a small effect size. The female group was specifically better at keeping their ring finger still when the other fingers moved compared to the male group (large effect size). Further, the female group also displayed fewer speed peaks than the male group (with a large effect size), indicating generally enhanced finger movement control compared to the male group. Similar to II and SI outcomes, the female group had fewer speed peaks primarily for the ring finger compared to the male group. The most prominent findings in relation to sex were shown when analyzing the effect of vision of the hands on movement trajectory distances. These analyses showed that only the movement trajectories of the female group differed significantly between the visual conditions, where trajectories were generally longer when vision of the hands was occluded. More specifically, the effect of vision for the female group was prominent for their dominant hand which moved an average of 13 mm longer when visual monitoring was prevented. These differences were specifically evident for the middle (21 mm longer in the no-vision condition) and ring (13 mm longer in the no-vision condition) fingers. In addition, other results show that IIs and SIs were significantly higher in the visual condition), but the associated effect sizes were however small (Tables 3, 4; Figs. 2, 3). As no statistically significant side differences were apparent in the II, SI nor SP outcomes the greater distance of the dominant hands fingers for the female group during the no-vision condition do not appear to exert an obvious negative influence on the movement independence and control measures used here. Nevertheless, of course we cannot conclude that the increase in trajectory length in the no-vision condition for the female group has a negative impact on the movement control measures used here. This as indices potentially could be higher if trajectory distances were equal across the conditions for the female group. The phenomenon of increased movement trajectory distance for the dominant hand fingers of females when visual monitoring is not possible needs to be replicated and further investigated for a better understanding. Taken together, in a task requiring no manipulation and where finger/hand dimensionality was controlled for, the female group showed evidence of increased individual finger movement control compared to the male group. The cause of these sex differences is unclear, but a possible explanation for the results of the current study could be that the female participants tended to participate in a higher frequency of, and possibly greater diversity in, fine manipulative activities than the male participants, which could positively affect the development of neuromuscular control processes (Table 2).

### Task-specific finger motor control—relations to individual finger movements

As in other studies using similar individuation calculations (Häger-Ross and Schieber 2000; Lang and Schieber 2004), the thumb was the most independent finger, both in terms of producing isolated movements (II) and remaining still when the other fingers of the hand moved (SI). As previously proposed (Häger-Ross and Schieber 2000; Lang and Schieber 2004), it is likely that the superior independence of the thumb is due to its greater muscular independence and to an increased neuromuscular control in relation to its importance in gripping. In support of this argument, the muscular anatomy of the thumb greatly contributes to it having the highest degrees of freedom of all the fingers, which in theory could make it a candidate for poor movement control. This is however not the case, as it had both the highest IIs and SIs as well as the lowest SDs. The thumb was also the finger with the most speed peaks, which could be a reflection of its higher degrees of freedom. The underlying explanation is not obvious, but could perhaps be related to the more individual muscle control of the thumb and its’ particular role in prehension. A greater number of velocity-based changes (i.e., speed peaks) would generally be regarded as an indication of poor movement control. Although the thumb moved at the same speed as the other fingers, the number of speed peaks and the II did not correlate (see Table 2). It is possible that these measures reflect either different aspects of movement control or are a reflection of the measurement characteristics where the II is a spatially derived cross-finger estimate and the SP is a within-finger temporal measure.

The index finger also showed high independence as indicated both by high II and high SI. The values were, however, not as high as compared to previous studies (Häger-Ross and Schieber 2000; Lang and Schieber 2004), in which it performed on par with the thumb. Interestingly, in our study, the index finger had significantly fewer speed peaks than all of the other fingers, indicating a high level of control. Humans rarely use their fingers independently, but when they do it is most often the index finger and the thumb acting in opposition in various functional fine motor tasks. Hence, the high level of control over the thumb and index finger has both biomechanical and neural underpinnings. The middle, ring and little fingers had similar IIs that were substantially lower than for the thumb and index finger. SI was also the highest for the thumb, index and for the little finger, with the ring and middle finger having the lowest SI. A similar pattern of performance was shown in the study by Häger-Ross and Scheiber (2000), who related the coupling observed to neuromuscular and anatomical constraints.

## Strengths and limitations

Although age did not correlate significantly with any of the outcome variables in the current study, including cohorts of younger participants would extend our knowledge of the effects of vision of the hands and sex on task performance. A strength of this study is that we collected 3D kinematics which made it possible to optimize the movement plane prior to calculations of II and SI. This procedure reduces possible errors that might occur if motion is measured in a fixed plane and if the person performing the test has rotated the hand so that part of the movement occurs outside the fixed plane. Also, the thumb has an additional movement plane compared to the other fingers, which is accounted for when using this 3D approach. Although the results obtained were similar to previous studies using 2D recordings, albeit with younger samples than in the current study (Häger-Ross and Schieber 2000; Lang 2004), the ability to optimize the movement plane should be regarded as beneficial as it improves data reliability. Although out of scope in the current study future studies should aim to investigate how temporal and spatial aspects are interrelated in finger movement control. 3D sampling should be a useful methodological approach to such investigations given its high spatial and temporal resolution. In addition, synchronized gaze recordings would add further information into the role vision has in guiding or correcting movements. The most consistent findings were related to differences in performance between the sexes. However, given the relatively low number of participants, we advise that these results should be taken with some caution. The stability of these results must be tested in larger samples where we also suggest that dexterity and, as here, fine motor experience is measured.

## Conclusions

The present study shows a small benefit of visual monitoring of the hands during a non-manipulative finger movement task, thus suggesting that visual feedback has only a minor influence on individual finger movement in healthy middle-aged and older individuals. A consistent finding was that females in general, for a task that did not require object interaction or manipulation, showed greater movement control of their fingers than males. This is a novel finding that adds important information to previous research where systematic sex differences in hand size have been shown to influence results in favor of females. Given the novelty of this finding and the relatively low number of participants in the current study it is important to perform replication studies including larger study groups.

## Data Availability

Data will be made available upon reasonable request.
